# Science of omics: a molecular space odyssey

**DOI:** 10.1113/EP092777

**Published:** 2025-04-05

**Authors:** Salomé Coppens, Christophe Hirtz, Margaux Vignon, Damian M. Bailey

**Affiliations:** ^1^ LBPC‐PPC University of Montpellier, CHU Montpellier, INM INSERM Montpellier France; ^2^ Department of Periodontology, Dental Faculty University of Montpellier Montpellier France; ^3^ Neurovascular Research Laboratory, Faculty of Life Sciences and Education University of South Wales Pontypridd UK

1

Space exploration has entered a transformative era, with missions to the Moon and Mars testing the limits of resilience in the most extreme environment humans have ever encountered. Astronauts will have to face the full force of the space ‘exposome’, an emergent concept that reflects the cumulative sum of all environmental hazards encountered during spaceflight: cosmic radiation, isolation and confinement, distance from Earth, hostile/closed environments and altered gravity fields (Bailey, 2025). Although integrative physiology offers a systems‐level understanding of how the body adapts to these extreme conditions, the burgeoning field of space omics (Figure 1) provides important molecular insight (Overbey et al., 2024). Space omics, integrating genomics, transcriptomics, proteomics, metabolomics and epigenomics, holds the potential to revolutionize astronaut health monitoring, risk stratification and future refinement/development of personalized countermeasures (Figure 2; Fernandez‐Gonzalo et al., 2025).

**FIGURE 1:**
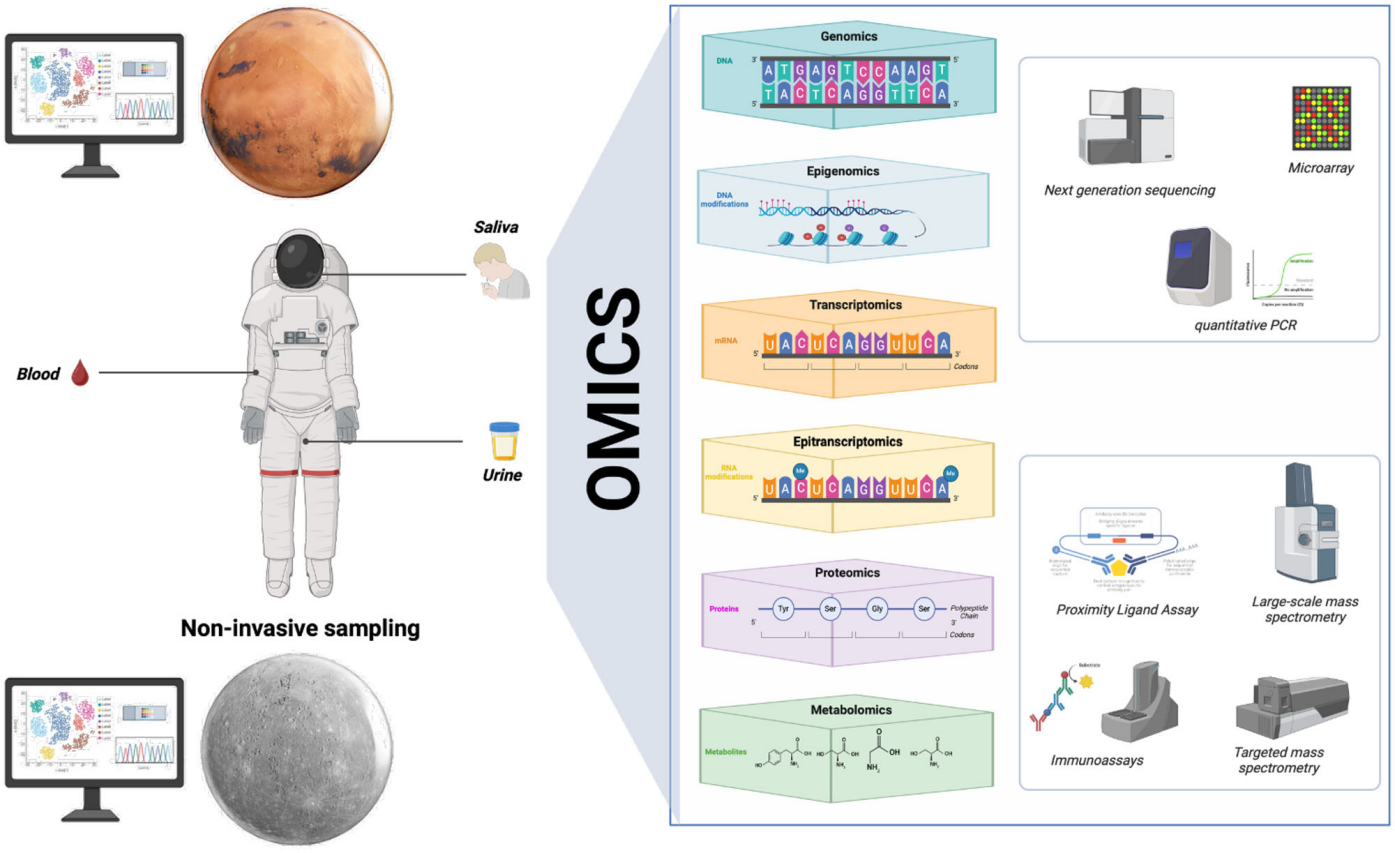
Human space multi‐omics approaches highlighting high‐throughput sequencing methods and technologies.

**FIGURE 2:**
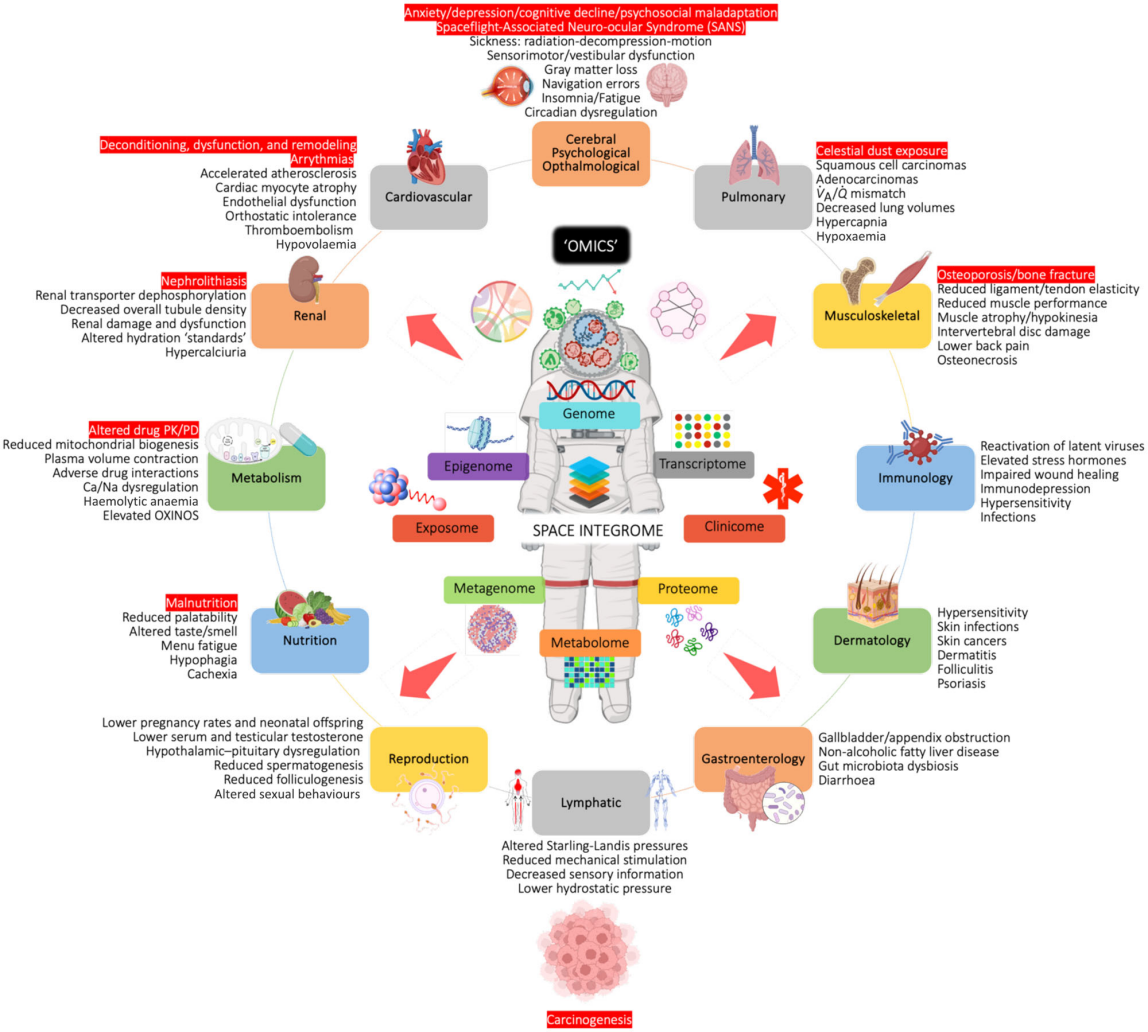
‘Omics’ insight into the space integrome. Organ‐specific health risks associated with the cumulative physiological impact incurred during exposure to the multitude of environmental stressors that collectively constitute the space ‘exposome’. Omics technologies have the potential to provide unique insights to inform an astronaut's personalized space ‘integrome’ and better inform spaceflight risk stratification to develop more effective, personalized countermeasures. Note that health risks highlighted red reflect those defined by the NASA Human Research Program [outlined within the HRP Integrated Research Plan (NASA, 2023)] with the highest priority based on likelihood of occurrence and severity of (adverse) consequences for crew health and performance during exploration‐class missions beyond low Earth orbit (i.e. cis‐lunar space, lunar surface operations, lunar outpost and Mars exploration) (Romero & Francisco, 2020). Abbreviations: OXINOS, oxidative–inflammatory–nitrosative stress; PK/PD, pharmacokinetics/pharmacodynamics; SANS, spaceflight‐associated neuro‐ocular syndrome; ${\dot{V}}_{A}/\dot{Q}$, ventilation/perfusion. Created with BioRender.com.

However, multiple challenges need to be addressed before space omics can unleash its full integrative potential for the benefit of human spaceflight. In the present editorial, we briefly highlight the transformative potential of space omics, the challenges it faces and the broader implications for both space exploration and terrestrial medicine.

Space omics aims to decipher the molecular alterations incurred during spaceflight, providing a deeper understanding of how the human body responds to the space exposome. A pioneering effort in this field was the NASA Twins Study, which investigated the molecular differences between astronaut Scott Kelly, who spent nearly a year aboard the International Space Station (ISS), and his twin brother, Mark Kelly, who remained on Earth. This research revealed spaceflight‐induced changes in gene expression, immune function, telomere length and microbiome composition, providing unique insight into the physiological implications of long‐duration spaceflight (Garrett‐Bakelman et al., 2019).

By harnessing these findings, space omics has the potential to drive personalized medicine for astronauts. Identifying genetic predispositions to a multitude of responses that collectively comprise the space ‘integrome’ (Bailey, 2025), including radiation sensitivity, bone density loss, cardiopulmonary/cerebrovascular risks and immune dysfunction, can facilitate the development of targeted, tailored countermeasures (Antonsen et al., 2023; Crucian et al., 2018; Overbey et al., 2024). This allows for proactive intervention, optimizing the well‐being of astronauts during extended missions. Another crucial area of investigation involves epigenetic adaptation, where space omics can help to determine whether heritable epigenetic modifications arise in response to long‐duration missions, an essential consideration for intergenerational space travel (Luxton et al., 2020). Epitranscriptomics can prove equally insightful given recent evidence for increased *N* ^6^‐methyladenosine modification (Grigorev et al., 2024).

Recent advances in omics technologies have significantly improved the sensitivity and precision of molecular analyses, enabling the detection of minute biological changes with unprecedented accuracy. Next‐generation sequencing has revolutionized genomics and transcriptomics by allowing high‐throughput analysis of genetic material, while mass spectrometry has dramatically improved proteomics and metabolomics, identifying thousands of molecular signatures from minuscule biological samples. Recent innovations have also led to the development of ultra‐sensitive sampling techniques, such as dried blood spots, capillary blood sampling, saliva collection and urine analysis, minimizing the need for invasive procedures (Gertz et al., 2020; Rutter et al., 2024).

Mass spectrometry has revolutionized the study of biological changes in the body by enabling the high‐throughput identification and quantification of biomolecules in a multitude of media. This approach is particularly valuable for understanding how environmental factors alter the proteome, providing insights into biomarkers that can inform personalized medicine. Dried blood spots and saliva have emerged as convenient, non‐invasive sampling methods for monitoring these proteomic changes, from which we can identify thousands of molecules. Dried blood spots technology allows astronauts to collect and store small blood samples without the need for bulky refrigeration equipment, ensuring sample stability until they can be batch processed post‐mission in a specialist laboratory. It offers a stable and easily transportable alternative to traditional blood collection, making it ideal for longitudinal studies and remote sampling (Vialaret et al., 2024). Likewise, saliva is increasingly sampled and has emerged as a viable alternative to blood sampling, because it contains biomarkers reflecting stress, immune response and metabolic changes (Vignon et al., 2023). The combination of mass spectrometry with these minimally invasive sampling techniques expands the possibilities for biomarker discovery, early detection and real‐time health monitoring.

Our collective ability to obtain reliable molecular data from micro‐sampling methods is particularly advantageous for spaceflight, where traditional blood collection methods are impractical owing to limited medical resources and the logistical constraints of handling fluids in microgravity. These advances in space‐compatible sampling are paving the way for real‐time health monitoring and personalized countermeasure development, bringing us closer to achieving sustainable and safe(r) human space exploration (Cope et al., 2022; Rutter et al., 2024).

Although space omics is primarily focused on astronaut health, its applications extend beyond space exploration, with translational benefits for medicine and human health on Earth. Insights gained from this research could revolutionize precision medicine by informing tailored treatment plans in fields such as oncology, cardiology and age‐related diseases, such as neurodegeneration. Additionally, spaceflight accelerates biological ageing, making it a valuable model for studying related mechanisms of ageing and potentially unlocking new anti‐ageing interventions for terrestrial populations (Camera et al., 2024; Kern & Siew, 2025). Furthermore, the modulation of the immune system during spaceflight provides insights into immune dysregulation, which could aid in the development of novel therapies for autoimmune diseases and inflammatory disorders (Crucian et al., 2018). Nutrigenomic findings from space omics research might also optimize personalized nutrition strategies, allowing individuals to tailor their diets based on genetic and metabolic profiles (Pittia et al., 2023). Beyond healthcare, space omics can inform medical care in extreme environments, benefitting professionals working in deep‐sea diving, Arctic research and disaster‐stricken regions where medical resources are scarce (Pirotta et al., 2022).

Despite its unrivalled potential, space omics faces several challenges that must be addressed to ensure its full integration into human spaceflight. One major limitation relates to the small sample size of spaceflight studies. With <10 individuals typically participating in space omics research, statistical power is invariably limited, making it difficult to validate biomarkers and detect consistent space stress‐induced pathways. Individual variability further complicates reproducibility, necessitating advanced statistical methods, longitudinal studies, and the integration of data from multiple missions and spaceflight analogues (Ray et al., 2019).

Additionally, sample collection, storage and transport conditions present logistical hurdles. Space studies often require trained personnel for sampling procedures, particularly for blood or cerebrospinal fluid collection. Saliva is often chosen as a more accessible alternative, but challenges remain regarding storage and transportation, because space environments often lack freezers or refrigerators, necessitating innovative preservation techniques (Rutter et al., 2024).

Another critical issue relates to the complexity of data interpretation. Space omics generates vast datasets that require sophisticated artificial intelligence‐driven analytics to extract meaningful insights. Distinguishing relevant biological signals from background noise in response to the space exposome is a formidable challenge. Ethical and privacy concerns must also be addressed, particularly concerning genetic profiling and data ownership (Rutter et al., 2024). Finally, translational barriers remain, because current pharmacogenomic and nutrigenomic approaches remain largely experimental in space settings (Mason et al., 2024).

As humanity ventures deeper into space, space omics will play an invaluable role in preserving crew health and performance. The integration of artificial intelligence‐driven diagnostics, wearable biosensors and gene‐based interventions could redefine how medical care is approached both in space and on Earth (Scott et al., 2023). Space omics will be essential for long‐term space colonization efforts, ensuring that humans can thrive and not merely survive in extraterrestrial environments. If outposts are established on the Moon, Mars or exoplanets, understanding how our physiology adapts to the unprecedented, cumulative stress imposed by the space exposome will be crucial for interstellar exploration. The journey to the stars is not only about exploration, but also about harnessing translational knowledge to benefit all of humanity.

## AUTHOR CONTRIBUTIONS

Damian M. Bailey conceived the original idea and, with Salomé Coppens and Christophe Hirtz, wrote the first draft of the manuscript. Salomé Coppens, Christophe Hirtz, Margaux Vignon and Damian M. Bailey revised the manuscript. Salomé Coppens, Christophe Hirtz, Margaux Vignon and Damian M. Bailey approved the final version submitted for publication and agree to be accountable for all aspects of the work in ensuring that questions related to the accuracy or integrity of any part of the work are appropriately investigated and resolved. All persons designated as authors qualify for authorship, and all those who qualify for authorship are listed.

## CONFLICT OF INTEREST

D.M.B. is Editor‐in‐Chief of *Experimental Physiology*, Chair of the Life Sciences Working Group, member of the Human Spaceflight and Exploration Science Advisory Committee to the European Space Agency, member of the Space Exploration Advisory Committee to the UK and Swedish National Space Agencies and member of the National Cardiovascular Network for Wales and South‐East Wales Vascular Network. D.M.B. is also affiliated to Bexorg, Inc. (USA) focused on the technological development of novel biomarkers of cerebral bioenergetic function and structural damage in humans.

## FUNDING INFORMATION

D.M.B. is supported by a Royal Society Wolfson Research Fellowship (grant no. WM170007).
